# Indents

**Published:** 1916-12

**Authors:** 


### INDENTS

**☞ Brief Articles of Technical or Scientific Value will receive notice and comment. Contributions invited.**

*The Pathologic Oral Cavity.* The field for the pathologist and bacteriologist is so large that its extent can be merely outlined. His aid is indispensable in the diagnosis of the constituency of saliva, in ptyalism, inflammations of the salivary glands, in ranula, in stomatitis of any kind, catarrhalis, nicotiana, alcoholia, apthosa and ulcerosa. He should be able to diagnose definitely any intoxications of the oral tissues, by acids, alkalies, iodin, bromin, phosphorus, arsenic, antimony, bismuth, mercury, alcohol, morphia, opium, heroin, cocain. He will render a reliable verdict on diabetes, uremia, nephritis, hemophilia, scurvy, anemia, leukemia; on infectious diseases such as fevers, anginas, acute articular rheumatism, mumps, influenza, erysipelas, typhus, cholera, croup, tetanus, lyssa, actinomycosis, tuberculosis; on myocarditis, endocarditis, pericarditis, aneurysm; on gastric disturbances including gastric ulcer and cancer; on the presence in the urine of albumin, sugar, blood, pus, indican; on measles, scarlet fever, diphtheria, noma in children; on alopecia, erythema, herpes, eczema, psoriasis, ichthyosis, lupus, lepra, skleridermia, urticaria, glossitis, actinomycosis, sporotrichosis, blastomycosis or mycosis fungoides in adults; on the innumerable phenomena of syphilis in its various stages as evidenced in the mouth; on diseases of pregnancy, eyes, nose, and throat; finally on occupational diseases of the mouth as produced by temperature changes, dust of all kinds, drugs, metals, etc.

In the face of this multiplicity of diseases any one of which the dentist is liable to meet in his daily practice, and which he frequently is the first to have an opportunity to see, it is manifest that the dentist's duties, if he would discharge them conscientiously, are so manifold that his time and skill are taxed beyond reasonable capacity. Yet a faulty diagnosis or neglect of the seriousness of the condition confronting him might justly bring condemnation upon his head, which he could avoid by intelligent and broad-minded co-operation with the pathologist and bacteriologist. As Gildersleeve remarks: "Mistakes in diagnosis were in a way excusable before we had at our disposal the data which enable us to call to our aid clinical laboratory methods. The fact that a practitioner does not possess laboratory facilities is not a plausible excuse. Every dental and medical practitioner should possess a clinical laboratory outfit and be so trained as to be able to employ such methods for the proper handling of his cases, or he should call to his aid those who are devoting their energies toward the conducting of clinical laboratories, and who are skilled in this branch." Specialization is more and more coming to its own, and the dentist who will intelligently avail himself of the opportunities offered in his profession will bring credit upon himself and his profession, and earn full moral and material recognition from his grateful patients.—Dr. F. K. Ream in N. J. *Dental Journal.*

*To remove Model from Articulator.* A safe and quick way to remove a model from the articulator is to grip the articulator in a bench vise, and cut through the plaster with a small hand-saw. This plan is especially suitable for the removal of lower models, which are so easily broken. If sawed off at the right depth no paring is necessary before flasking—Dr. Brownlee in *Dominion Journal.*

*Early Orthodontic Treatment.* As to the cause of malocclusion, I would not undertake to even venture an opinion, nor to open up this broad avenue of discussion. I know that different authors have different ideas of the causes. Some claim that it may be possible to trace the origin of malocclusion even as far back as the intra-uterine life, others claim that it is possible to prove that malocclusion is due entirely to the improper care of the child's mouth in early infancy, but on one point they all agree, and that is the necessity of stimulating the growth of the maxilla. This is too often overlooked, resulting in undeveloped jaws. From my own observations I have not found a child with perfectly developed lungs where there is either a constricted palatal arch, or a prognathic lower maxilla, and if my observations are correct the importance of correcting malocclusion as early as it appears becomes obligatory.—Dr. L. P. Torrence in *Dental Review.*

*Emetine in Pyorrhea.* In early cases of pyorrhea, emetine is valuable, inasmuch as it will destroy amoeba, and thus remove one source of irritation, while repair is going on. When cure is effected, micro-organisms can not enter healthy tissue; consequently, a new mechanical irritation, or lowered vitality, must be established to permit of their entrance. In advanced cases emetine is valuable, as an adjunct treatment, by removing one source of irritation, even though its administration must be repeated from time to time. In these cases a constant supervision is necessary, and emetine should be employed in some form of application, just as antiseptics, astringents or any other medications are used.—Dr. Julian Smith in *Western Journal.*

*The Care of Children's Teeth.* Some years ago, a dentist charged the writer very seriously and emphatically never to permit the teeth of his children-patients to be neglected; and this counsel has been faithfully heeded. It was with a good deal of astonishment that this writer read, recently, in the chairman's address before the section on diseases of children of the American Medical Association (*Jour. Amer. Med. Assoc.,* 1916, July 29, p. 323) the assertion that indifference to the subject of children's teeth pervades both the medical and dental professions, and that, indeed, many members of the latter seem wofully ignorant of the great importance of conserving children's teeth.

It appears that many dentists refuse to accept children as patients for the purpose of trying to save their deciduous premolars or even the first permanent molars, on the ground that it is "not worth while." The excuse is, that fillings, inserted for preserving the deciduous teeth will fall out in a short time and, consequently, must be replaced; that early decay of the temporary teeth does not injure the children; and that malformation or malocclusion of the permanent teeth can later be corrected, if it occur in consequence of premature loss of the deciduous teeth.

It is surprising that such a specious excuse can either be made or accepted at the present juncture. There are many reasons, inmediate as well as remote, why children's teeth should receive the same painstaking attention which is accorded the permanent ones, and a failure to do this or a refusal on the part of the dentist to attempt to save the deciduous teeth until they fall out spontaneously is little short of criminal. We of the older generation, whose childhood was passed during the days when little attention was paid to baby-teeth because they would fall out anyway, know from sad experience the distress and pain which is suffered, not only from aching teeth, but also because of the unsightly appearance of the decaying teeth; for, children are not without vanity—and it is just and right that they should wish to look as well as possible.

Aside from the fact that the appearance of a little tot with decaying baby-teeth is anything but pleasant, it must not be forgotten that this condition may affect the permanent teeth; that it gives occasion for a faulty, insufficient, onesided use of the jaws and, in consequence, to its faulty development. The esthetic point of view arguing in favor of attending to every slightest anomaly and irregularity of the body is, by no means, of small importance and may become a factor in deciding the future fate of the child, especially the girl-child.

Of even greater importance is the fact that an insufficient use of the teeth, in the case of small children just as much as in that of older ones and of adults, may be responsible for digestive disturbances of various intensity and extensity, and which may lead to serious gastro-intestinal diseases, with all their possible sequels. But, most of all, should one pay attention to the care of the deciduous teeth on account of the ever-present bacterial flora in the mouth of the child, even of the youngster that has not yet lost its baby-teeth. If any anomaly exists in the small mouth, if, for instance, caries develops, a portal of entry is given for various bacteria, which may cause swelling of the cervical lymph-glands and from there may be carried into various organs of the body, where serious disturbances and pathological processes may be set up.

A great variety of pathogenic bacteria have been found in carious deciduous teeth, and in recent years the importance and significance of "focal infection" has been insisted upon, with great justice, by many investigators and clinicians. The care of the baby-teeth is, therefore, as much a wise investment for the preservation of health as is any other prophylactic measure, quite aside from the fact that much actual physical pain and mental distress will be spared to the little ones, which rotten teeth surely will cause them to suffer.—*Clinical Medicine.*

*Conservatism in Extractions.* Since the message was sent broadcast by a few investigators in the field of root pathology that apical foci of infection were responsible for the onset of chronic infections in remote areas of the body *thousands of teeth have been uselessly sacrificed.* It has been the experience of a number of practitioners, the writer included, that the conclusions of enthusiasts in the field are not *always* warranted by the clinical results secured in many chronic cases following the removal of presumably infected roots. Case after case could be summoned in support of the contention that root pathology in its bearing upon the development of systemic lesions is as yet in semi-obscurity and that the so-called focus of infection upon or adjacent to a tooth root is not to be construed in every instance as the only source of a generalized disturbance. This conclusion is reached after having observed a weighty number of cases in which no appreciable improvement could be observed after months of patient waiting. We have seen patients regain their health after the extraction of infected roots or the surgical treatment of their apices; we have also seen patients from whose mouths as many as ten teeth have been extracted in the hope of eliminating a deep-seated infection with no other result than the loss of their masticatory organs.

We believe that to a large extent these negative results can be traced to a misconception of the pathologic significance of certain shadows in radiographic films or plates and to the unwarranted advice so liberally dispensed that tooth roots presenting what is erroneously interpreted as an active focus of infection should be at once sacrificed to the forceps. Clinical results are flatly contradicting the assumption that every shadow upon the root of a tooth in a radiograph is a focus of infection, and laboratory experiments in those same cases are likewise giving negative results. A shadow on the apex of a root may indicate one of several things, herein including the filling up of the cancellated spaces in the alveolar bone by a proliferation of the soft tissue which normally fills the cancellated spaces. If as the result of an involvement of the alveolar bone, following an infection emanating from the pulp or pericemental membrane, some of the cancellated spaces are destroyed, as occurs in osteo-myelitis, following, or simultaneous with, an inflammatory proliferation of the myelitic substance (the substance contained in the cancellated spaces), a dark shadow will be brought out upon the radiograph even though all bacterial elements be absent, *i. e.*—the density of the area has been materially decreased by the filling in of the enlarged cancellated spaces by soft tissues. Again, in the case of a chronic infection of the peridental membrane which has spread to the alveolar process, causing the loss of osseous substance with perhaps practically no attempt at obliteration of the space by organic tissue, a dark shadow will be shown on the plate even after complete eradication of the infection.

Another misconception of root pathology is in the tendency to regard dark shadows upon radiographs as evidence of the presence of granulomata, viz., tumors or enlargements of the peridental membrane made up of a large number of embryonic connective tissues containing within its meshes epithelial elements or again made up entirely of connective tissue. The number of teeth which we have examined after extraction which upon previous radiographic examination showed fairly large blackened areas adjacent to the apical region, leads us to the conclusion that the presence of granulomata is the exception rather than the rule.

A "Stop—Look—Listen" sign is appearing in the dental horizon and the leaden forceps of the Temple of Apollo at Delphi is again casting its shadow. Valuable as unquestionably the X-ray has become in the hands of the radiographer-pathologist, equally as dangerous it is becoming in the absence of a complete system of diagnosis which should include the history of the case; the clinical examination, laboratory findings and the X-ray. Complete reliance on the radiograph is, at the present time at least, not warranted as the sole means of diagnosis. *It is not the X-ray machine alone that the dental office needs, as much as it does the X-ray machine plus correct pathologic interpretation of lesions of the teeth and adjacent structures.*—Editorial in *Pacific Gazette.*

*Failures I Have Had.* Has any one ever read a paper before a dental society upon the subject of this editorial? If they have I must confess that in fifteen years of dental journal reading and society attendance I have never seen or heard one.

None of us like to confess that we have failures, but nevertheless we all meet with them in greater or lesser numbers and degree. Some one has truly said that "we learn by our mistakes" and failures surely come within this category. If we individually can profit or learn in this way why would not a recital of the facts prove of benefit to some other practitioner. Rare, indeed, is the paper in which a writer makes any illusion to anything but the successful operations he has performed, and naturally so, for it is far more pleasant to tell of the successful than the unsuccessful occurrences, but candidly the writer is tired of this constant presentation of papers, essays, talks, and the like, all proclaiming nothing but the fact that the author or lecturer is a one hundred percent. perfect operator in everything which he undertakes. At least, that is the impression most of them give.

I have often wondered if these ultra successful essayists gave any thought at all to the effect which their assertion of their own infallibility had upon many of their hearers. Let me illustrate: A while ago a paper dealing with root treatment and filling was presented before a dental society. The essayist claimed to succeed in filling all roots completely, with not a come back or any ensuing trouble of any kind. He had radiographs to prove it, and they did show apparently perfect fillings. After the meeting a group was discussing the subject and two or three of the men declared that they had conscientiously endeavored to follow the essayist's technique in many cases, but with indifferent results, or, at best, with a number of failures. They were discouraged or disgusted and were unanimous in their belief that the essayist was more or less of a nature faker and they had no faith or belief in his methods. Still the methods advocated by this writer were thoroughly scientific according to present day standards, and probably in his hands gave a large proportion of successful operations, but how much greater would have been his influence for good had he confessed that he also met with canals which he could not fill perfectly and that he, occasionally, broke a broach in a root and that he at times had some of the other unpleasant experiences which every practitioner of dentistry meets with.

The above example may be just as pertinently applied to the crown and bridge worker, the orthodontist, the inlay enthusiast, or the specialist in artificial dentures.

T. R. or some other prominent man has said that "the person who never makes mistakes never makes anything," and some one else remarks that "to have tried and failed is better than not to have tried at all," and as "honesty is the best policy," and "an honest confession is good for the soul," there are well-founded grounds upon which an essayist may stand and, with perfect propriety, confess that, skillful operator that he is, mistakes and failures sometimes crown his most carefully performed operations.—Editorial, N. J. *Dental Journal.*

*Gold Plating in Dentistry.* Pure gold next to porcelain and pure platinum is the least irritating to the oral tissues of any of the restorative materials used in reparative dentistry. Bridges and gold plates are usually made of alloyed gold. However, lower karat then 18 karat, high grade gold solder should never be used in dental prosthesis. In many mouths, the oral secretions and gases act readily on alloyed metal and it becomes unsightly and unclean, besides often causing a disagreeable metallic taste, which is augmented if there are alloy fillings in some of the remaining teeth.

It is my practice to gold plate the piece after it is thoroughly finished and highly polished before placing it in the mouth, this can be done in various ways but the method to use now is a very simple one, using the Orvis Perfected Gold Plating Solution which can be purchased at any good dental depot. Follow the directions closely, being especially careful to thoroughly cleanse the piece before immersing it in the solution. The finished piece will plate beautifully and the results are most gratifying. —G. Walter Dittmar in *Dental Review.*

*Cavity Varnish.* Dissolve some copal in equal parts of alcohol and chloroform; add one equal volume of hydro-napthol, and the product will be a very adhesive and strongly antiseptic varnish, free from all caustic properties.—*Stomatologist.*

*Sir William Osler on Wounds and Disease in War.* Sir William Osler has contributed to the *Quarterly Review* an interesting survey of wounds and disease in the war. He states that for the first ten months of the war the killed numbered 50,432, the equivalent of the annual toll taken in England alone by one bacillus, that of tuberculosis. Of the 153,980 wounded, up to date 60 per cent. have returned to duty. The returns for disease are not yet available, but when the analysis is completed, he thinks that we shall find that coughs and colds, pneumonia, rheumatic fever, bronchitis and muscular rheumatism, particularly what is known as "trench rheumatism," have been responsible for a large portion of the sickness. More suppurating wounds have been seen by British surgeons in ten months than in forty years since Lister's methods became general. The nature of the commoner wounds due to shell and shrapnel favors infection, and in spite of early first-aid dressings, the germs are carried far into the tissues, and suppuration is inevitable. Trench warfare can never be aseptic. The uniforms of the men are caked with mud, which in highly cultivated districts always contains dangerous microorganisms, so that any wound, however slight, unless treated immediately, becomes infected. The one bright feature has been the frequency with which the high-velocity bullet sterilizes its course so that from many of the perforating wounds of the chest, abdomen and limbs there is recovery without suppuration. In the early days of the war, no feature was more distressing than the number of cases of tetanus; but medical science provided a remedy. Orders were received that at first-aid stations all wounded were to receive a protective antitetanic vaccination. No single feature of the campaign has been more gratifying in its results. Since every wounded man has received at the first dressing station a protective inoculation, the disease has been checked.—*Journal, A. M. A.*

*Root Canal Work* Root canal work is divisible into two major classes. Where the pulp is found alive, but exposed, the dentist should be able to remove it and fill the canals without causing or inviting infection in ninety per cent. of cases. Where the tooth is already infected, the acute cases should be curable, provided no previous practitioner has mutilated the original canal with drills. Many chronic infections are curable likewise, either by treatment through the apex or by root amputation. Finally all diseased teeth that can not be cured should be extracted.—Dr. R. Ottolengui in *Items of Interest.*

*Novocain made in England.* Owing to the war it has been impracticable to secure novocain in England from Germany, where it has been made. The English Comptroller of Patents has granted permission to one English chemical concern to make it and will probably grant a similar permit to other drug manufacturers who can show themselves competent to manufacture it correctly. Chemists all over England are taking up the manufacture of chemicals and drugs heretofore made in Germany only, but under present conditions can not be obtained. The Government seems to have waived all patent rights. Undoubtedly England will in a short time be making all such goods within her own territory. It may be of great benefit to this country to have a source of supply that is accessible, but it is probable that when the war is over we shall secure our drugs from Germany again, probably at much lower prices because of the English competition.

*A Dental Automobile for the English Army.* A new automobile dental surgery, costing nearly $5,000, has been presented to the Army by the Royal Automobile Club, through the Red Cross Society. It will be in service close behind the firing lines in France. It is completely equipped with all accessories for taking care of the soldiers' dental needs. It is not designed especially to take care of the accidental injuries from gunshot wounds, etc., so much as to keep the soldier free from tooth disturbances which would hamper their usefulness or effectiveness. The car was inspected by the King and Queen at Buckingham Palace and met the hearty approval of the King.

*Succinimid of Mercury in the Treatment of Pyorrhea Alveolaris.* On page 957 of the August *Cosmos*, Geo. E. Cox, D.D.S., of Wilmington, Del., relates an unfortunate experience with succinimid of mercury in the treatment of pyorrhea.

The treatment of pyorrhea with succinimid of mercury in my practice has produced 100 per cent. of cures, and I find no other treatment to equal it. For the past eight years I have been treating this disease and have tried every treatment which seemed worthy, but have found none to equal the above.

Violent pain and later sloughing of the tissues at the point of injection, as mentioned by Dr. Cox, are typical of cases in which the injection has not been made sufficiently deep. I am sure that is the cause of the after-effects mentioned rather than the result of infection. In my opinion this injection can not be made deeply enough at any point except in the gluteal muscle. There the injection can be made two and one-half inches deep almost without pain, and the results are always satisfactory—of course, provided an aseptic operation has been performed. In one case I had a physician make three injections for me of 5/5 grain in the deltoid muscle, and the results were all that could be desired, but I have since insisted that the injections be made in the gluteal muscle. When I have a lady patient who, like the doctor's, is too modest to have the injection made by her physician at that point, I simply use emetin, or make the injection myself.

Of course, I always give a thorough local treatment and insist on the patient's massaging the gums regularly with the fingers, the massaging movement being from the teeth toward the gums, but with the tooth brush the movement is from the gums toward the teeth.

I am keeping a careful record of all patients treated with succinimid of mercury, and I find that Dr. P. G. White has not claimed too much for this method. E. D. McAdory, M. D., of this city, has worked in co-operation with me in this treatment, making most of the injections. In this connection I may add that more co-operation between the two professions would be particularly beneficial to both, especially since we are learning how many bodily ills may arise not only from this disease, but from unclean oral cavities.—G. W. Bledsoe, D.D.S., in *Dental Cosmos.*

*Perforation of Root-canal Walls.* Perforation of the canal wall is an unfortunate accident, but one which may happen in the best regulated dental practice. When this occurs, the canal should be rinsed of all debris and a dressing made of a non-irritating antiseptic —such as a mixture of oil of cloves and wood creosote—the teeth closed, and allowed to rest for several days. This dressing may be changed several times until it is impossible to dry the place of perforation by means of alcohol and warm air. If this spot is then wetted with oil of cajuput, or better still, Dr. Callahans solution of rosin in chloroform, the excess of liquid removed, and a small, thin wafer of slightly warmed gutta percha be applied thereon and gently carried to place with a suitable plugger, this awkward point of weakness should be permanently protected. Of course the foregoing presupposes that the opening is accessible and visible. A perforation near the apical end of the canal is difficult of treatment, and requires the most delicate skill and good judgment. Under strict asepsis, the gutta percha filling should obliterate that point of weakness.—W. R. Dunning, *Dental Digest.*

*To Obtund Dentin.* It is good practice to sterilize the cavity with alcohol before desensitizing with "iodrenalcain," for both excavating and immediate pulp removal. With this method of procedure, in a large number of cases, perfect anesthesia can be obtained almost instantaneously, and remains long enough to give ample time for completing the operation in hand. Results are most gratifying to both patient and operator. Teeth that were hypersensitive and tender to stress of mastication previous to operation become comfortable and useful members.—Elmer E. Lampert, *Dental Review.*

*Iodin as a Douche for Teeth.* *To the Editor:*—I have found marked benefit from the application of tincture of iodin, five per cent. solution, to the gums around the roots of the teeth. Do you know of any good substitute, as the iodin discolors the teeth. Please do not publish my name.—W. F.

ANSWER.—Black, in his work on "Dental Pathology," recommends that the patient be taught to douche the gums twice daily with physiologic sodium chlorid solution. A

rubber bulb syringe with a tolerably large nozzle is advised. This is to be held in such position that the stream will pass into and wash out the space between the edge of the gums and the teeth. In this way inflammation, which would otherwise result from accumulation of bacteria and their products, may be prevented. Black also states that more than half of all inflammations of the gums are due to slight traumatisms of various kinds, due to such causes as the lodgment of food between teeth, rough edges of fillings, crowns, etc.—*Journal A. M. A.*

*Repairing an Inlay Margin.* Sometimes for good and sufficient reasons It becomes necessary to seat an inlay that is good in every respect excepting a slight flaw or fault in one of the margins. This can very easily be fixed with a little gold foil. Have the surface of the inlay clean, grasp it firmly in a pair of tweezers, flux slightly and heat in a blue flame to a dull red. While at this heat a small pellet of uncondensed foil is stuck to the part of the margin that is shy. Then the inlay is placed in the cavity, allowing the foil to condense against the cavity wall. When the inlay has been seated with cement the margins will be without the former flaw.—*Pacific Gazette.*

*Synthetic Crowns.* Dr. Land has had a number of forms of teeth made in celluloid, and very thin; somewhat like one-half of a medicine capsule, only shaped like incisors, cuspids and bicuspids. The root is prepared and a celluloid form selected of proper size and shape. Next a pin is cemented into the root. Then the celluloid form is filled with synthetic cement, and while the cement is still plastic it is forced to place, the pin protruding from the root being pressed into the plastic cement. The celluloid is left on for twenty-four hours, and when removed leaves a synthetic cement tooth, highly polished and really very good in appearance if a good color is chosen.—*Dental Items of Interest.*

*To Give a Facing a Light Gray or Blue Shade.* To give a facing the light gray or blue shade at the tip when the body and the gingival should be of the yellow shade, can be very easily accomplished by laying a piece of 1/1000 platinum foil between the facing and the gold backing in the incisal third. Thus the reflection becomes a grayish color which would otherwise have been yellowish due to the presence of the gold back.

*Bad Teeth and Ill Health.* The department of health estimated that in 1915 the actual cost to the citizens of Chicago of three contagious diseases—measles, diphtheria and scarlet fever—among children was more than six and one-half million dollars. Nor does that take into consideration the number of deaths. All of these diseases are preventable. The germs were communicated mostly by bad teeth.

"This was demonstrated by a circumstance in one of the schools while our commission was studying the problem. There had been an epidemic of scarlet fever, and it seemed impossible to stamp it out effectually. It was then decided to allow no children who had scarlet fever to return to school until their teeth had been examined and treated. This measure stopped the sporadic epidemics in that school."

And if there still remain doubters, after reading this testimony, let us turn to a place where the "disease" that has to be cured is not so easily handled—since its cause has not yet been isolated; a place where so-called criminals are being "treated"—for that really is what they are doing in an eastern penitentiary.

In that institution a modern dentist's chair has, for more than two years, been a regular part of the prison equipment. And a skilled dentist is on the pay-roll. More than that, tooth brushes and dentifrices have been added to the stock in the prison store.

The dentist does all necessary work without charge. Inmates who wish extra work done may get it at special rates. And while the use of the tooth brush is not compulsory—the class there dealt with is better led than driven—it has spread until now there are few who are not as regular in this toilet practice as in washing their hands and faces.

"The result has been little short of astonishing," said a high prison official lately. "Since the dentist began making regular visits to the institution, not only the health of the inmates, but their behavior, has improved. After long experience with men sent to prison, I am convinced that the misdeeds of such unfortunates may, in some measure, be traced to lack of proper care of the teeth and mouth. Our experience in the penitentiary has proved that there is a mental—and, therefore, moral—side to this, as well as a physical."—*Extract from paper in North American.*

*Plates Rather than Bridges.* Cheap and consequently septic bridgework should be counted as malpractice, and should give place to the clasped parital denture; and we believe that if the inventive geniuses in the dental profession will for a time apply their thoughts to the construction of clasps there will be evolved one or even several modes of procedure whereby the man with moderate means, the man who simply can not buy good bridgework and the good root canal work which must accompany it, will still be well served and be better off than were he to attempt to masticate food with less than the full complement of teeth.

In short, a renaissance in prosthodontia is due, and we prophesy that it will arrive and bring with it the next important progressive step in dentistry, the supplying of sane and sanitary dentures at a low cost and with a minimum of danger to the remaining natural organs.—Dr. Ottolengui in *Dental Items of Interest.*

